# Prevalence and factors associated to recurrent wheezing phenotypes in the NOVA lima asthma program

**DOI:** 10.1186/1939-4551-8-S1-A201

**Published:** 2015-04-08

**Authors:** Rosiléa Alves Silva, Laura Maria Belizario Facuri Lasmar

**Affiliations:** 1 Hospital Infantil João Paulo II – Fhemg, Brazil; 2 Universidade Federal De Minas Gerais, Brazil

## Background

Several studies on population basis have been carried out to determine prevalence and incidence of recurrent wheezing phenotypes and its predictor factors. Among those, factors have been described related to the pre-natal, early life and environment characteristics.This article evaluates the prevalence and the associated factors of recurrent wheezing phenotypes in asthma control programs.

## Methods

Cross sectional study involving 374 patients with diagnosis of recurrent wheezing or asthma included in Nova Lima asthma program. Atopy and pulmonary function were investigated. The phenotypes have been classified into four groups. Group 1, patients started and stopped wheezing up to three years old. Groups 2 and 3, patients started wheezing before three years old and went up to and after six years old, respectively. Group 4, started wheezing after three years old and persisted in after six years old.

## Results

The 374 patients were classified as groups 1, 2, 3 and 4, with respectively 17, 4, 23, 5, 51, 9 and 7,2%. Passive maternal smoking was significantly more prevalent in the groups 3 (p<0,001) and 4 (p=0,02), comparing with group 1. Child passive smoking was significantly more prevalent among the groups 3 (p= 0,03) and 4 (p= 0,02) comparing to group 1. After multivariate analysis, parental asthma, passive maternal smoking and rhinitis with allergic sensitization were independent factors associated to the wheezing persistence after three years old.

## Conclusions

Approximately 41% of patients stopped wheezing up to six years old. Rhinitis with allergic sensitization, passive maternal smoking and parental asthma history increased the odds of wheezing after three years old. The recognition of these factors can contribute to the morbidity reduction and improvement of care quality.

